# A Randomized Placebo-Controlled Trial of Metformin for Frailty Prevention in Older Adults

**DOI:** 10.1093/geroni/igab046.2991

**Published:** 2021-12-17

**Authors:** Amir Tavabi, Chen-pin Wang, Joel Michalek, Tiffany Cortes, Ethan Leonard, Becky Powers, Nicolas Musi, Sara Espinoza

**Affiliations:** 1 University of Texas Health Science Center San Antonio, San Antonio, Texas, United States; 2 UT Health San Antonio, Texas, United States; 5 UT Health San Antonio - Barshop Institute, San Antonio, Texas, United States; 6 South Texas Veterans Health Care System, San Antonio, Texas, United States

## Abstract

Frailty is a progressive physical decline leading to higher morbidity and mortality in older adults. Previous studies have demonstrated shared mechanisms between insulin resistance, inflammation, and frailty. The purpose of this trial is to determine whether metformin prevents frailty in non-frail, community-dwelling older adults (≥65 years) with pre-diabetes, determined by 2-hour oral glucose tolerance test (OGTT). Frail individuals (Fried criteria) and those with renal impairment (glomerular filtration rate <45 mL/min) are excluded. Eligible participants are randomized to metformin or placebo and followed for two years. The primary outcome is frailty; secondary outcomes include physical function (short physical performance battery), systemic and skeletal muscle inflammation (plasma and muscle inflammatory markers), muscle insulin signaling (muscle biopsy), insulin sensitivity (insulin clamp), glucose tolerance (OGTT), and body composition (dual-energy x-ray absorptiometry) measurements. Participants are followed every 3 months for safety assessments, every 6 months for frailty assessment and OGTT, and every 12 months for muscle biopsy. Currently, 99 participants, including 53 (53.5%) male and 91 (91.9%) white, are active (54) or have completed the study (35). At baseline, mean age was 72.3 ± 5.5 years, body mass index was 30.7 ± 5.9 kg/m2, and Hemoglobin A1c was 5.73 ± 0.37%. Mean frailty score was 0.5 ± 0.6 and the proportion of non-frail and pre-frail participants were 58.6% (n = 58) and 41.5% (n = 41), respectively. Findings of this clinical trial may have future implications for the use of metformin in older adults with pre-diabetes in order to prevent the onset of frailty.

